# Genetic characteristic of coexisting of *mcr-1* and *bla*_NDM-5_ in *Escherichia coli* isolates from lesion-bearing animal organs

**DOI:** 10.3389/fmicb.2023.1116413

**Published:** 2023-03-15

**Authors:** Yungai Xiang, Zengyuan Liu, Guo Yu, Yuxia Song, Yan Li, Xujing Geng, Liying Ma, Junqing Guo, Li Tan, Pengju Chen

**Affiliations:** ^1^Department of Reproductive Medicine, The Second Affiliated Hospital of Zhengzhou University, Zhengzhou, Henan, China; ^2^College of Pharmacy, Shenzhen Technology University, Shenzhen, China; ^3^Henan Institute of Modern Chinese Veterinary Medicine, Zhengzhou, Henan, China; ^4^Shandong Xindehui Biotechnology Company Ltd., Yuncheng, Shandong, China

**Keywords:** *Escherichia coli*, *mcr-1*, *bla*_NDM-5_, lesions-bearing, chromosome

## Abstract

The coexistence of *mcr-1* and *bla*_NDM-5_ in the plasmid of *Escherichia coli* has been widely reported and such strains have been mainly isolated from animal and human feces. However, few reports have focused on the genetic diversity of *mcr-1*-carrying chromosomes and *bla*_NDM-5_-carrying plasmids in *E. coli* isolates from lesion-bearing animal organs. This study investigated the genetic characteristics of chromosome-mediated *mcr-1* and plasmid-mediated *bla*_NDM-5_ in *E. coli* isolated from lesion-bearing animal organs. Nine *mcr-1*- and *bla*_NDM-5_-positive *E. coli* strains (MNPECs) showed extensive drug resistance (XDR). The predominant clonal complexes (CC) mainly belonged to CC156, CC10, and CC165 from the 56 MNEPCs (including nine strains in this study) retrieved from the literature. These strains were widely distributed in China, and originated from pig fecal samples, human stool/urine samples as well as intestinal contents of chicken. Two transconjugants harboring *bla*_NDM-5_ gene were also successfully obtained from two donors (J-8 and N-14) and this transfer increased the MIC for meropenem by 256 times. However, conjugative transfer of *mcr-1* gene failed. Both J-8 and N-14 strains contained point mutations associated with quinolone resistance and more than three types of AMR genes, including the *mcr-1* gene on the chromosome and the *bla*_NDM-5_ gene on the IncX3-type plasmid. The genetic structure of *mcr-1* located on the chromosome was an intact Tn*6330*, and *bla*_NDM-5_-carrying IncX3-type plasmid was IS*Ab125*-IS*5*-*bla*_NDM-5_-*bleO*-*trpF*-*tat*-*cutA*-IS*26* gene cassette. Moreover, differences between chromosomes included additional partial sequence of phage integrated into host genome and the different genes associated with *O*-antigen synthesis.

## Introduction

Antimicrobial resistance currently represents one of the most serious global threats to human and animal health as it can cause the rapid evolution and emergence of multi-drug-resistant (MDR), extensively drug-resistant (XDR) and even pan drug- resistant (PDR) gram-negative pathogens (Martin et al., 2020). One example is the case of *Escherichia coli*, the most common pathogen within the *Enterobacteriaceae* family, since it can not only cause severe infection in humans and animals, but is also a major reservoir of virulence/antimicrobial resistance genes (ARGs) (Aslam et al., 2018; Poirel et al., 2018). Indeed, over the past decades, *E. coli* has acquired various ARGs through horizontal gene transfer, a process which enabled the pathogen to exhibit extensive drug resistance. Such resistance is often manifested in the form of extended-spectrum beta-lactamase (ESBL) activity that results from different acquired drug resistance genes (ESBL; Hordijk et al., 2020).

Carbapenems, colistin, and tigecycline are currently known to be the last-resort antibiotics for the treatment of MDR gram-negative pathogens (Mckenna, 2013; Nordmann and Poirel, 2014). Initially, with the increasing prevalence of infections caused by ESBL-producing *Enterobacteriaceae* is increasing, carbapenems were used as a significantly-effective remedy against these pathogens (Sheu et al., 2019). However, the frequent misuse of this class of antibiotics eventually led to the emergence of carbapenem-resistant *Enterobacteriaceae* (CRE). For instance, New Delhi metallo-β-lactamase 1 (*bla*_NDM-1_), a new carbapenem resistance gene, was first reported in 2009 and exhibited resistance to almost all of the currently available β-lactam antibiotics (Yong et al., 2009; Zhou et al., 2017b). The *bla*_NDM-1_ gene responsible for this type of resistance subsequently disseminated rapidly around the world (Kumarasamy et al., 2010) before evolving into 44 known NDM variants (*bla*_NDM-1_ to *bla*_NDM-43_).^1^ Of these, *bla*_NDM-1_ and *bla*_NDM-5_ represent the most common variants (Wu et al., 2019), with the latter exhibiting a higher-level of carbapenemase activity than *bla*_NDM-1_ as a result of amino acid substitutions at position 88 (Val → Leu) and 154 (Met→Leu; Hornsey et al., 2011; Wu et al., 2019).

In addition to carbapenems, colistin has also been used as a last-resort effective antibiotic to treat severe human and animal infections caused by CRE (Kempf et al., 2016; Poirel et al., 2017). However, despite its wide use in veterinary medicine as a feed additive, the application of colistin in human medicine was more restrictive due to its neurotoxic and nephrotoxic effects (Landman et al., 2008; Kempf et al., 2013). With the emergence of ESBL and CRE bacteria that were resistant to most classes of available antibiotics as well as the shortage of new antimicrobials, colistin was subsequently reconsidered as a viable therapeutic option (Nation and Li, 2009; Karaiskos et al., 2017) but this gradually led to the evolution and emergence of colistin resistance.

The mechanism of bacterial resistance to colistin was previously shown to be due to point mutations in two-component systems (TCSs), namely the PhoP/PhoQ and PmrA/PmrB, located on chromosomes. As such, colistin resistance was considered to be spreadable only through vertical transmission (Olaitan et al., 2014). However, *mcr-1*, the mobilized colistin resistance gene, allowed colistin resistance to be spread through plasmids, with this additional mode of transmission being first reported in *E. coli* isolated from pigs and humans in China in 2015 (Liu et al., 2016). *Escherichia coli* strains carrying the *mcr-1* gene have since been described globally from a wide range of sources, including humans, animals, foods and the environment (Lv et al., 2016; Yang et al., 2016; Yao et al., 2016; Zhou et al., 2017a).

The emergence of coexisting *mcr-1* and *bla*_NDM-5_ genes in gram-negative bacteria can therefore pose a serious threat for the treatment of MDR or XDR infections, especially due to availability of limited treatment options. In fact, *E. coli* containing both genes have already been isolated from humans, animals, the environment and retail meat, with these strains being distributed in different ST types, such as ST10, ST156, ST167 etc. (Mediavilla et al., 2016; Sun et al., 2016; Long et al., 2019; Liu et al., 2021a). Moreover, while the *mcr-1* gene is commonly carried by three types of plasmids (IncI2-, IncX4-, and IncHI2-type), *bla*_NDM-5_ is mainly found on the IncX3-type plasmid (Kong et al., 2017; Ho et al., 2018; Jiang et al., 2020). However, chromosomal *mcr-1* remains very rare compared with the plasmid-bearing one. At the same time, in *E. coli*, the coexistence of *mcr-1* and *bla*_NDM-5_ on chromosomes and plasmids respectively, is rarely found, with many reports instead describing the coexistence of both on *E. coli* plasmids (Mediavilla et al., 2016; Yang et al., 2016; Zhang et al., 2017; Ho et al., 2018).

In the present study, *E. coli* strains containing chromosomal *mcr-1* as well as the plasmid-bearing *bla*_NDM-5_ were isolated. The sequence type distribution and the genetic characteristics of these strains were then studied. It is expected that the findings may contribute to the understanding of the epidemiology, evolutionary mechanisms and genomic characteristics of the *mcr-1* and *bla*_NDM-5_ genes.

## Materials and methods

### Isolation and identification of MNEPCs

Samples were obtained from lesion-bearing organs of swine, chicken and dairy cow between 2016 and 2019 in Yangling, Shannxi, China. The sampling was performed as our previously described (Liu et al., 2021b). MacConkey agar (MAC) plates containing 2 μg/mL of colistin and 2 μg/mL of meropenem was used for the isolation of MNEPCs. The species were further identified by the amplification and sequencing of 16S rRNA. Then, colistin and carbapenem resistance genes were detected by amplification and sequencing of the *mcr-1* and *bla*_NDM-5_ genes (Nordmann et al., 2011; Liu et al., 2016).

### Antimicrobial susceptibility testing of MNEPCs

Antimicrobial susceptibility testing (AST) was performed by broth microdilution method as our previously described (Liu et al., 2021b). Briefly, 0.5 McFarland inoculum suspensions were diluted 1:100 to a final inoculum density of 10^6^ CFU/ml. Ampicillin (AMP), ceftazidime (CAZ), cefotaxime (CTX), aztreonam (AZM), tetracycline (TET), doxycycline (DOX), ciprofloxacin (CIP), enrofloxacin (ENR), gentamicin (GEN), sulfamethoxazole (SMZ), fosfomycin (FOS), florfenicol (FFC), colistin (COL), and meropenem (MEM) (Solarbio, Beijing, China) were selected to determine the minimum inhibitory concentrations (MICs) of MNPECs. The MICs were recorded as the lowest concentrations of antimicrobials in the wells, where no visible bacteria growth was observed. *Escherichia coli* ATCC 25922 was used as standard reference strains.

### Multi-locus sequence typing of MNEPCs

Genomic DNA of MNEPCs was extracted using Wizard® Genomic DNA Purification Kit (Promega) according to manufacturer’s protocol and sequenced using next-generation sequencing on a NovaSeq PE150 at the Beijing Novogene Bioinformatics Technology Co., Ltd. Purified genomic DNA was quantified by fluorometer and high quality DNA (OD260/280 = 1.8–2.0) was used to do further research. DNA samples were sheared into 400–500 bp fragments using a Covaris M220 Focused Acoustic Shearer following manufacture’s protocol. Illumina sequencing libraries were prepared from the sheared fragments using the NEXTflex™ Rapid DNA-Seq Kit and then were used for paired-end Illumina sequencing (2 × 150 bp) on an Illumina HiSeq X Ten machine.

The raw data were then submitted to Enterobase^2^ and the results of MLST analysis were automatically obtained using the Achtman scheme. To obtain clean data, the raw sequences were filtered prior to genome assembly using SOAPdenovo, SPAdes and Abyss software. The resulting assembly was then integrated with CISA software and the least scaffolds was selected for follow-up analysis. In addition, MNEPCs that co-carried *mcr-1* and *bla*_NDM-5_ were extracted from published literature (2016–2022) in PUBMED. A minimum spanning tree was eventually generated using PHYLOViz 2.0 software to analyze the distribution of sequence type of MNPECs (Francisco et al., 2012; Nascimento et al., 2017).

### Mating experiments between MNEPCs

The ability of the *mcr-1* and *bla*_NDM-5_ genes to be transferred was evaluated using the broth-mating assay. For this purpose, two MNPECs were selected as donor strains and were mixed in a ratio of 1:3 along with the recipient strain C600. The mixtures were then incubated at 37°C and 180 r/min for 4 h before being spread on MAC plate (2 mg/ml streptomycin +2 μg/mL colistin or 2 mg/mL streptomycin +2 μg/mL meropenem). The plates were eventually incubated at 37°C for 24 h. In addition, the MICs of transconjugants were also determined using broth microdilution method as described above.

### Whole-genome sequencing analysis of MNPECs

Our studies have shown that the similarity of the PFGE patterns of 9 MNPECs resistant to meropenem and colistin is 100% (Data not shown), meanwhile, 9 MNPECs were of the same ST type. Therefore, one representative strain J-8 was selected for WGS. The sequencing of strain J-8 was performed by Shanghai Majorbio Pharmaceutical Technology to produce 2 × 150 bp paired-end reads (MiSeq, Illumina, San Diego, CA, USA) as well as long reads (Pacific Biosciences, Menlo Park, CA, United States). The two sets of reads were then assembled using Unicycler.

This was followed by the automatic annotation of J-8’s genomes using the rapid annotations using subsystems technology (RAST).^3^ Plasmid replicon types, acquired resistance genes, chromosomal mutations, virulence genes and FimH type were then identified with the PlasmidFinder 2.1,^4^ ResFinder 4.1,^5^ Resistance Gene Identifier (RGI),^6^ VirulenceFinder 2.0^7^ and FimTyper 1.0,^8^ respectively. In addition, conjugal transfer components and type IV secretion systems (T4SS) on the plasmids and chromosomes were analyzed with oriTfinder,^9^ while insertion sequence (IS) elements, transposons (Tn) and integrons (In) were identified using ISfinder,^10^ INTEGRALL^11^ and MobileElement Finder v1.0.3.^12^ Phylogroups were eventually analyzed with the ClermonTyping tool.^13^ The above bioinformatics analyses were also repeated to assemble the sequencing date for N-14.

### Genetic analysis of the *mcr-1* and *bla*_NDM-5_ genes

The genetic environment of chromosomes carrying the *mcr-1* gene and plasmids carrying the *bla*_NDM-5_ gene were analyzed using BLAST Ring Image Generator (BRIG) and EasyFig tools (v2.3).

### Nucleotide sequence accession numbers

The complete genomes of J-8 were deposited in Genbank under the accession number CP047002 (J-8 chromosome), CP047003 (pTEM), CP047004 (pCTX), CP047005 (pfosA3), CP047006 (pNDM), CP047007 (p0), CP047008 (p1), CP047009 (p2), while that of N-14 was deposited with BioProject accession number PRJNA892028.

## Results

### AST of MNPECs

After isolating bacterial strains from 109 samples, nine of the MNPECs were selected for subsequent analysis based on coexisting *mcr-1* and *bla*_NDM-5_ genes in *E. coli*. Of these, eight strains were from the intestine and liver of chickens, and one strain was from the lavage fluid of dairy cow with endometritis (Table 1). The MICs of the nine MNPECs against 14 antimicrobials were as shown in Table 1. In particular, nine of the MNPECs showed resistance to all tested antibiotics and exhibited XDR.

**Table 1:** The MICs of nine MNPECs against 14 antimicrobials.

Strain	Origin	Source	MIC (μg/mL)													
			AMP	CAZ	CTX	AZM	TET	DOX	CIP	ENR	GEN	SMZ	FOS	FFC	COL	MEM
J-1	Chicken	Intestine	>512 ^R^	>512 ^R^	>512 ^R^	128 ^R^	128 ^R^	16 ^R^	128 ^R^	256 ^R^	128 ^R^	>512 ^R^	>512 ^R^	512 ^R^	4 ^R^	64 ^R^
J-2	Chicken	Intestine	>512 ^R^	>512 ^R^	>512 ^R^	256 ^R^	128 ^R^	16 ^R^	128 ^R^	256 ^R^	128 ^R^	>512 ^R^	>512 ^R^	512 ^R^	4 ^R^	32 ^R^
J-3	Chicken	Intestine	>512 ^R^	>512 ^R^	>512 ^R^	128 ^R^	128 ^R^	32 ^R^	256 ^R^	256 ^R^	128 ^R^	>512 ^R^	>512 ^R^	512 ^R^	4 ^R^	64 ^R^
J-4	Chicken	Intestine	>512 ^R^	>512 ^R^	>512 ^R^	256 ^R^	128 ^R^	16 ^R^	128 ^R^	256 ^R^	128 ^R^	>512 ^R^	>512 ^R^	512 ^R^	4 ^R^	128 ^R^
J-5	Chicken	Intestine	>512 ^R^	>512 ^R^	>512 ^R^	512 ^R^	256 ^R^	16 ^R^	128 ^R^	256 ^R^	128 ^R^	>512 ^R^	>512 ^R^	512 ^R^	4 ^R^	128 ^R^
J-6	Chicken	Intestine	>512 ^R^	>512 ^R^	>512 ^R^	256 ^R^	128 ^R^	16 ^R^	128 ^R^	256 ^R^	256 ^R^	>512 ^R^	>512 ^R^	512 ^R^	4 ^R^	64 ^R^
J-7	Chicken	Intestine	>512 ^R^	>512 ^R^	>512 ^R^	256 ^R^	128 ^R^	16 ^R^	128 ^R^	256 ^R^	256 ^R^	>512 ^R^	>512 ^R^	>512^R^	4 ^R^	32 ^R^
J-8	Chicken	Liver	>512 ^R^	>512 ^R^	>512 ^R^	256 ^R^	128 ^R^	16 ^R^	128 ^R^	256 ^R^	256 ^R^	>512 ^R^	>512 ^R^	512 ^R^	4 ^R^	64 ^R^
N-14	Dairy cow	Uterus	>512 ^R^	>512 ^R^	>512 ^R^	256 ^R^	128 ^R^	16 ^R^	128 ^R^	256 ^R^	256 ^R^	>512 ^R^	>512 ^R^	>512^R^	4 ^R^	64 ^R^
ATCC 25,922	─	─	<0.25^S^	<0.25^S^	<0.25^S^	<0.25^S^	<0.25^S^	<0.25^S^	<0.25^S^	<0.25^S^	<0.25^S^	2	<0.25^S^	<0.25^S^	<0.25^S^	<0.25^S^

Ampicillin (AMP), ceftazidime (CAZ), cefotaxime (CTX), aztreonam (AZM), tetracycline (TET), doxycycline (DOX), ciprofloxacin (CIP), enrofloxacin (ENR), gentamicin (GEN), sulfamethoxazole (SMZ), fosfomycin (FOS), florfenicol (FFC), colistin (COL), meropenem (MEM). S, susceptible; R, resistant.

### Diversity of MNPECs’ distribution

**Figure 1:**
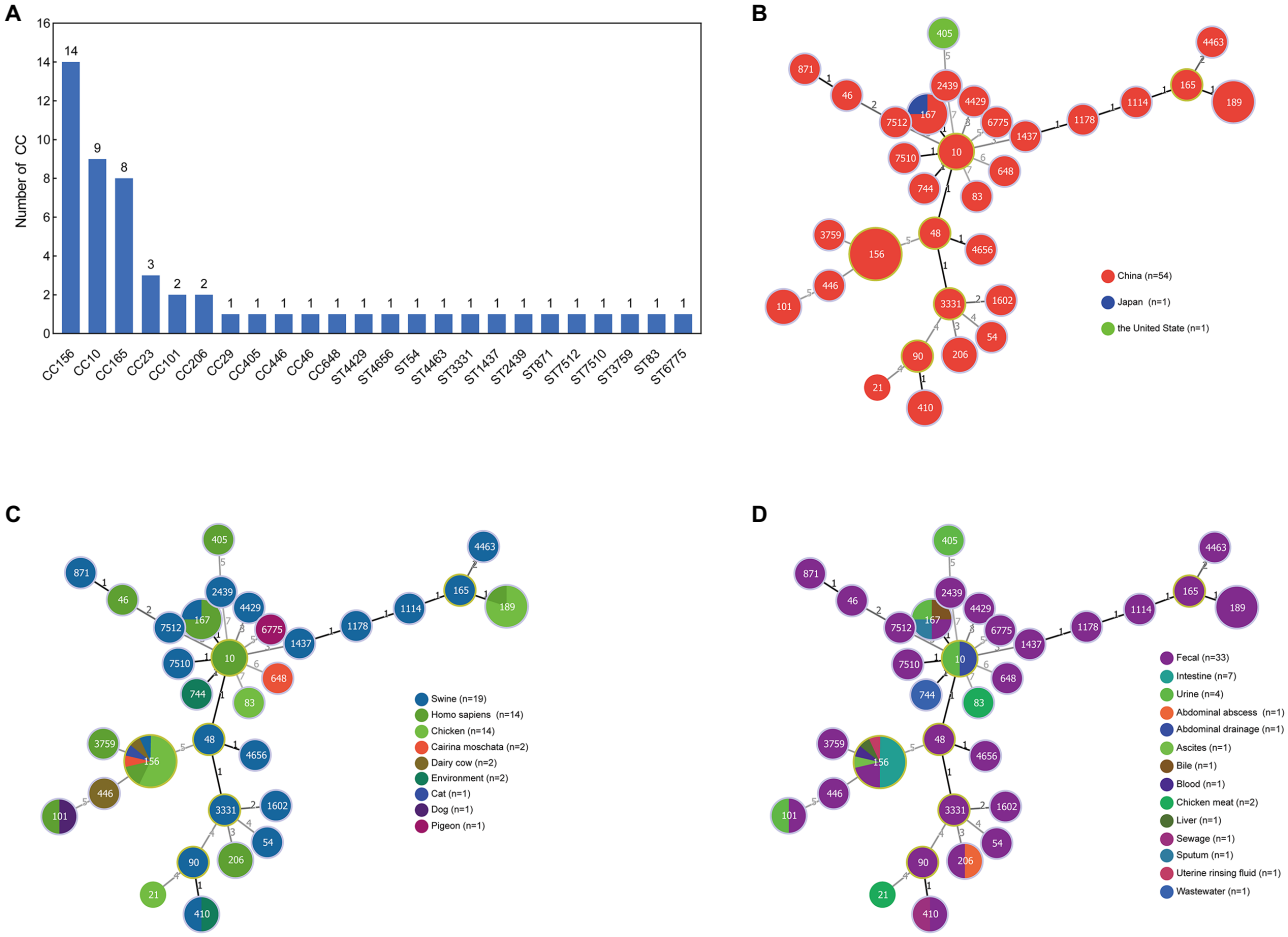
Analysis of sequence type (ST), country, host and source of 56 MNPECs. **(A)** Distribution of sequence type. Each node represents a distinct country **(B)**, host **(C)** and source **(D)**. The size of the circle represents the number of isolates with the same ST profile. The number on the line represents the count of the locus variants for ST.

A total of 56 MNEPCs were collected from literature (Supplementary Table S1). All of the nine isolated MNEPCs belong to ST156, and were used, along with the collected strains, for sequence typing as well as an analysis of the host and sources. The sequence types of the MNPECs strains were mainly distributed in CC156 (ST156; *n* = 14), CC10 (ST10, ST167, ST48, ST744, and ST1602; *n* = 9) and CC165 (ST189, ST1178, and ST165; *n* = 8; Figure 1A). Furthermore, in terms of geographical distribution, it was found that MNPECs were mainly distributed in China (*n* = 54; Figure 1B), with the hosts being mainly swine (*n* = 19), humans (*n* = 14) and chickens (*n* = 14; Figure 1C). At the same time, their main source were feces (*n* = 33), intestinal contents (*n* = 7) and urine (*n* = 4; Figure 1D). MLST analysis further indicated that CC156, CC10, and CC165 were the dominant clones. Thus, altogether, the results suggest that MNPECs are widely distributed in China, where they are mainly present in pig fecal samples, human stool and urine samples as well as the intestinal content of chicken. These findings also indicate that the coexistence of the *mcr-1* and *bla*_NDM-5_ genes in *E. coli* tend to be very common in animals and humans in China, with CC156 (ST156) being the dominant clone.

### Transferability of the *mcr-1* and *bla*_NDM-5_ genes in MNPECs

Two of the MNPECs (J-8 and N-14) were selected for the broth mating experiment based on the antimicrobial resistance spectrum, sequence type, host and source of the eight MNPECs in the present study. The *bla*_NDM-5_ gene in both MNPECs was successfully transferred to the recipient strain, with the two resulting transconjugants showing the same MIC for meropenem (64 μg/mL) as the donors, although the MIC for meropenem was increased by 256 times compared with the recipient strain C600 (0.25 μg/mL; Table 2). Besides, the transconjugants also exhibited resistance to AMP, CAZ, CTX, and AZM. However, a transconjugant of the *mcr-1* gene, transferred to the recipient strain, could not be obtained. The mating assay also indicated that the plasmid carrying *bla*_NDM-5_ could be successfully transferred to C600 and showed extensive resistance to β-lactam antibiotics. In contrast, the conjugative transfer of the *mcr-1* gene failed.

**Table 2:** The MICs for MPECs and transconjugants.

Strain	MIC (μg/mL)													
	MEM	COL	AMP	CAZ	CTX	AZM	TET	DOX	CIP	ENR	GEN	SMZ	FOS	FFC
EC600	<0.25 (S)	<0.25 (S)	2 (S)	<0.25 (S)	<0.25 (S)	<0.25 (S)	2 (S)	1 (S)	<0.25 (S)	<0.25 (S)	1 (S)	32 (S)	64 (S)	2 (S)
T-J-8^NMD^	64 (R)	<0.25 (S)	>512 (R)	>512 (R)	256 (R)	256 (R)	2 (S)	1 (S)	<0.25 (S)	<0.25 (S)	0.5 (S)	32 (S)	64 (S)	2 (S)
T-N-14^NDM^	64 (R)	<0.25 (S)	>512 (R)	>512 (R)	256 (Rc)	256 (R)	2 (S)	1 (S)	<0.25 (S)	<0.25 (S)	0.5 (S)	32 (S)	64 (S)	2 (S)

T represents the transconjugant; S, susceptible; R, resistant.

### Genetic characterization of MNPECs

Both *E. coli* J-8 and N-14 belonged to the phylogroup B1, and the *fimH* genotypes were *fimH1127*. The complete sequence of one chromosome and seven plasmids for J-8 were obtained through hybrid assembly based on next-generation sequencing and third-generation sequencing technology (Table 3). In this case, the genes *mcr-1* and *bla*_TEM-1B_ were located on J-8’s chromosome and four points mutations were found at the *gyrA* (S83L, D87N), *parC* (S80I) and *parE* (S458A) genes. At the same time, the chromosome also carried seven virulence genes, namely the *astA* (EAST-1 heat-stable toxin), *gad* (glutamate decarboxylase), *iss* (increased serum survival), *hlyE* (avian *E. coli* haemolysin), *lpfA* (long polar fimbriae), *papC* (outer membrane usher P fimbriae), *terC* (tellurium ion resistance protein). Strain J-8 also carried seven plasmids, of which four plasmid replicons were IncFIC (II), IncFIB, IncY and IncX3, and the rest were unknown. The plasmid pTEM carried nine antimicrobial resistance (AMR) genes: β-lactams (*bla*_TEM-1B_), aminoglycosides [*aadA2*, *aac(3)-IId*, *aph(3′)-Ia*], fluoroquinolones (*oqxA* and *oqxB*), sulfonamides (*dfrA*12), macrolides [*mph(A)*], peroxides (*sitABCD*). It also carried nine virulence genes: *cma* (colicin M), cvaC (microcin C), *hlyF* (hemolysin F), *iroN* (Enterobactin siderophore receptor protein), *iss*, *iucC* (aerobactin synthetase), *iutA* (ferric aerobactin receptor), *ompT* (outer membrane protease) and *sitA* (iron transport protein). On the other hand, the plasmid pCTX carried only one AMR gene: β-lactams (*bla*_CTX-M-55_), while the plasmid pfosA3 carried six AMR genes: aminoglycosides [*aph(6)-Id*, *aph(3″)-Ib*], tetracyclines [*tet(A)*], sulfonamides (*sul2*), fosfomycin (*fosA3*), amphenicols (*floR*). Finally, the plasmid pNDM carried two AMR genes: β-lactams (*bla*_NDM-5_) and bleomycin (*bleO*). Genome sequences of 98 scaffolds of N-14 were then obtained through next-generation sequencing technology. The four-point mutations and 19 AMR genes on the genome of strain N-14 were identical to those of strain J-8, with the plasmid replicons being also similar. However, the virulence genes *cma* and *cvaC* were not detected in strain N-14. Therefore, both strains J-8 and N-14 contained point mutations associated with quinolone resistance as well as more than three types of AMR genes, especially the *mcr-1* gene located on the chromosome and the *bla*_NDM-5_ gene located on the IncX3-type plasmid. The presence of both genes in the same strain conferred extensive drug resistance to the strains.

**Table 3:** Genomic features of two MNPECs.

Isolate name	FimH type	Phylogroup	Chromosome/plasmid	Size (bp)	Inc type(s)	Resistance gene/chromosomal point mutation	Virulence gene
J-8	*fimH1127*	B1	chr-J-8_1	4,834,034		*mcr-1.1*, *bla*_TEM-1B,_ *gyrA* (S83L, D87Y)	*astA, gad, iss, hlyE*, *lpfA, papC, terC*
			pTEM	116,304	FIB, FIC(FII)	*bla*_TEM-1B_, *aadA2*, *aac(3)-IId*, *aph(3′)-Ia*, *oqxA*, *oqxB*, *dfrA12*, *sitABCD*, *mph(A)*	*cma, cvaC, hlyF, iroN, iss, iucC, iutA, ompT, sitA*
			pCTX	112,016	FIB	*blaCTX-M-55*	—
			pfosA3	88,744	Y	*aph(6)-Id*, *aph(3″)-Ib*, *tet(A)*, *sul2*, *floR*, *fosA3*	—
			pNDM	46,161	X3	*bla*_NDM-5_, *ble*O	—
			p0	5,772	—	*—*	*—*
			p1	4,505	—	*—*	*—*
			p2	3,373	—	*—*	*—*
				
N-14	*fimH1127*	B1	Scaffold1-98	—	FIB, FIC(FII), Y, X3	*gyrA* (S83L, D87Y) *parC* (S80I) *mcr-1.1*, *bla*_TEM-1B_, *bla*_CTX-M-55_, *bla*_NDM-5_, *aadA2*, *aac(3)-IId*, *aph(3′)-Ia*, *aph(3″)-Ib*, *aph(6)-Id*, *tet(A)*, c*oqxA*, *oqxB*, *mph(A)*, *sul2, dfrA12*, *floR*, *fosA3*, *bleO*, *sitABCD*	*astA*, *iss*, *lpfA*, *hlyE*, *hlyF*, *papC*, *terC*, *iroN*, *iutA*, *iucC*, *ompT*, *sitA,*

*chr-*, chromosome; *p-*, plasmid; *Inc-*, incompatibility. Acquired resistance genes: colistin, *mcr-1*; β-lactam, *bla_TEM-1B_*, *bla_CTX-M-55_*, *bla_NDM-5_*; tetracycline, *tet(A)*; aminoglycosides, *aadA2*, *aac(3)-IId*, *aph(3′)-Ia*, *aph(3″)-Ib*, *aph(6)-Id*; fluoroquinolones, *gyrA [pS83L (Serine→Leucine)*; *D87N (Aspartic acid→Asparagine)]*, *parC [S80I(Serine→Isoleucine)]*, *parE [S458A(Serine→Alanine)]*, *oqxA*, *oqxB*; macrolides, *mph(A)*; sulfonamides/trimethoprim, *sul2*, *dfrA12*; phenicol, floR; fosfomycin, *fosA3*; peroxides, *sitABCD*.

### Analysis of the genetic environment of chromosomal *mcr-1*

**Figure 2:**
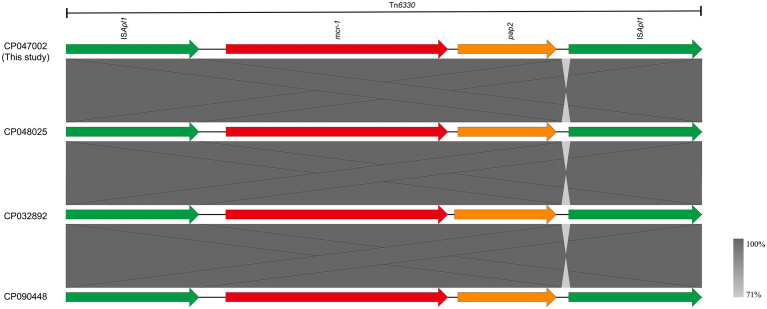
Genetic environment of *mcr-1*, located on the chromosome of *Escherichia coli* J-8 (CP047002), in this study and compared with GZEC065 (CP048025), SCEC020022 (CP032892), and PT62 (CP090448). Dark grey shading denotes regions of homology (>99% identity nucleotide identity).

The *mcr-1* gene in strain J-8, located on the chromosome (4,834,034 bp; GC content of 50.75%), was flanked by two intact IS*Apl1* elements, forming a composite transposon Tn*6330* (4,653 bp, IS*Apl1*-*mcr-1*-*pap2*-IS*Apl1*; Figure 2). Furthermore, this region (Tn*6330*) of J-8’s chromosome (CP047002) was highly similar to that of GZEC065 chromosome (CP048025, nucleotide coverage 100%; identity 99.98%), SCEC020022 chromosome (CP032892, nucleotide coverage 100%; identity 100%) and PT62 chromosome (CP090448, nucleotide coverage 100%; identity 100%). These results indicated that the genetic structure of *mcr-1* located on the chromosome was an intact Tn*6330* which was highly conserved in MNPECs.

### Chromosomal characteristics of MNPECs

**Figure 3:**
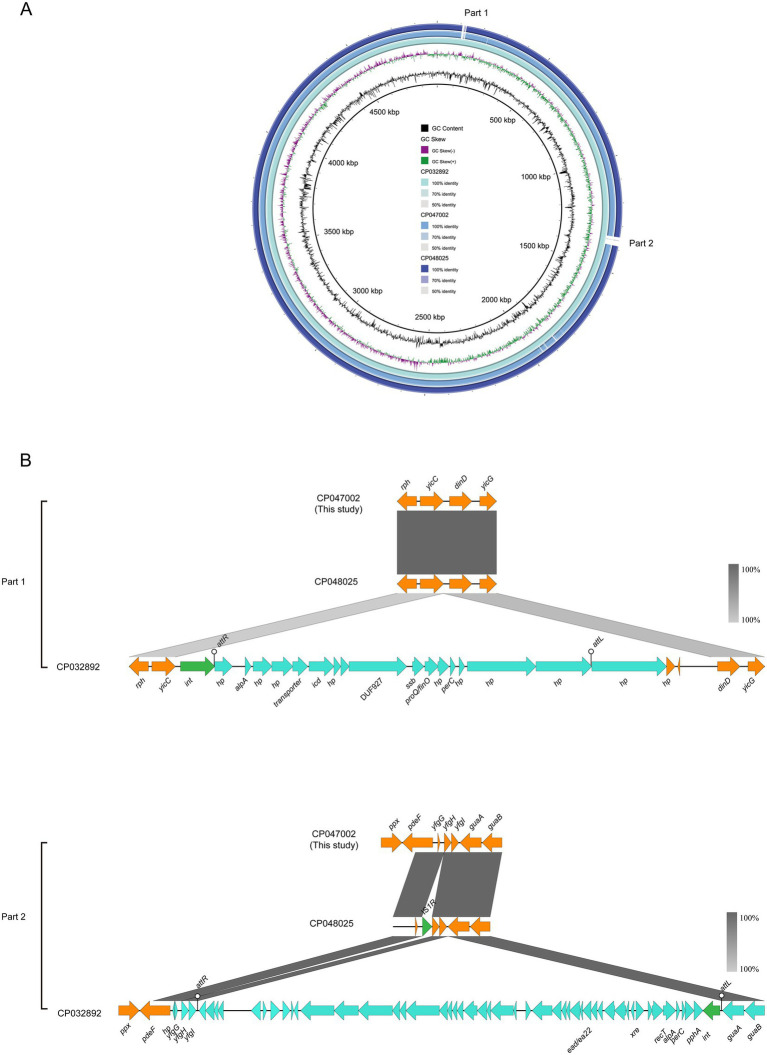
Comparative genomic analysis of *mcr-1*-carrying chromosomes of ST156 J-8 (CP047002, this study) compared with those of *Escherichia coli* ST156 SCEC020022 (CP032892), GZEC065 (CP048025) **(A)**. The chromosome of *E. coli* SCEC020022 was used as a reference sequence in this case. Colinear sequence comparison of Part 1 and Part 2 on the chromosome SCEC020022, along with chromosomes J-8 and GZEC065. Boxed arrows represent the position and transcriptional direction of ORFs. Dark grey shading denotes regions of homology (>99% identity nucleotide identity) **(B)**. Orange colors represent other genes; cyan colors represent the genes coding phage protein; green colors represent the genes coding integrase (*int*) and insertion sequence (IS).

**Figure 4:**
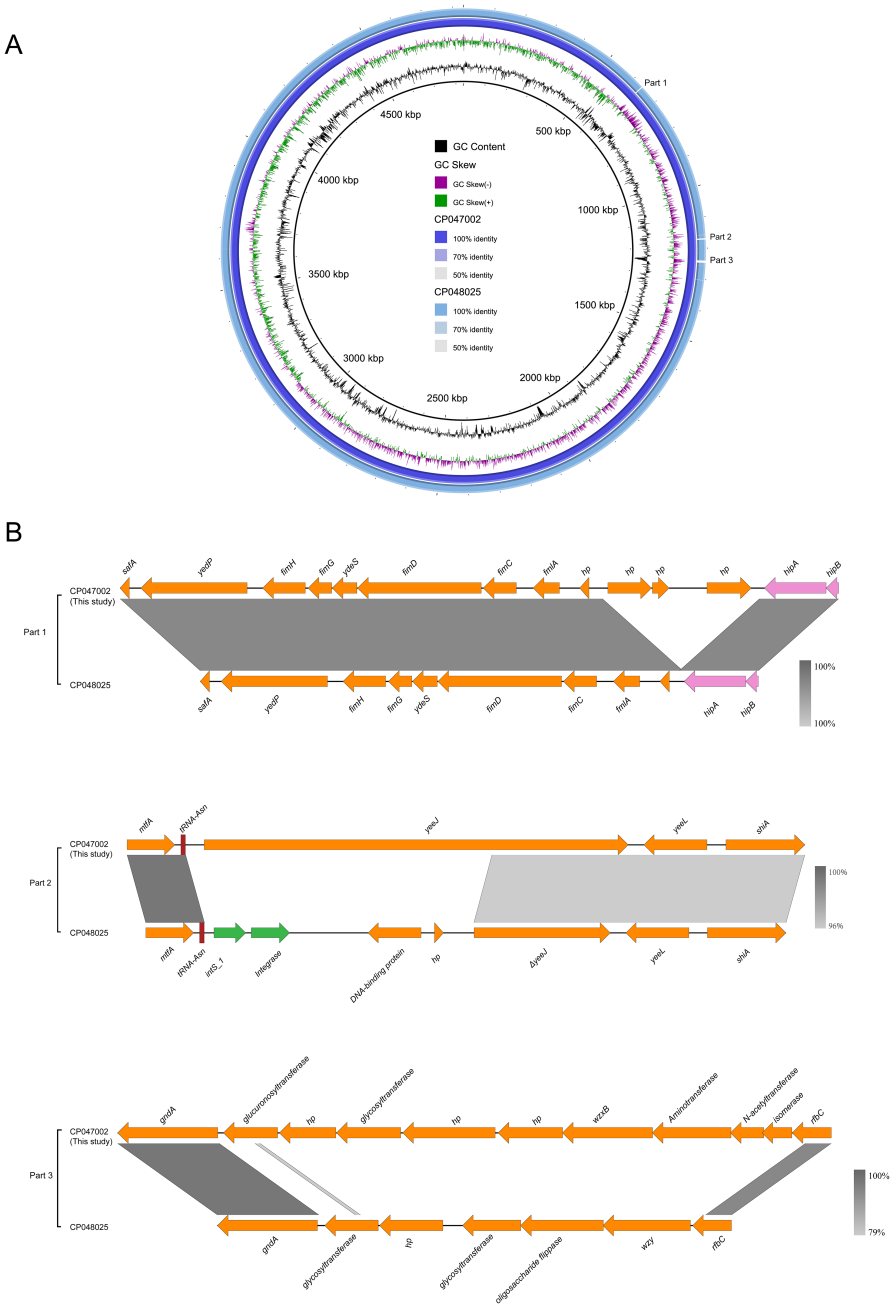
Comparative genomic analysis of *mcr-1*-carrying chromosome in ST156 J-8 (CP047002, this study) against *Escherichia coli* ST156 GZEC065 (CP048025) **(A)**. The chromosome of *E. coli* J-8 was used as a reference sequence. Colinear sequence comparison of Part 1, Part 2, and Part 3 with J-8’s and GZEC065’s chromosomes **(B)**. Boxed arrows represent the position and transcriptional direction of ORFs. Dark grey shading denotes regions of homology (>99% identity nucleotide identity). Orange colors represent other genes; pink colors represent the genes associated with type II toxin-antitoxin (TA) system; green colors represent the genes coding integrase (*int*).

Two *E. coli* ST156 strains carrying the *mcr-1* gene on the chromosome were selected for comparative genomics analysis with the J-8 chromosome. The latter was highly similar to that of the GZEC065 chromosome (CP048025, nucleotide coverage 100%; identity 99.90%) and the SCEC020022 chromosome (CP032892, nucleotide coverage 100%; identity 99.90%). Compared with the SCEC020022, the chromosomes of J-8 and GZEC065 lacked two distinct parts (Part 1 and Part 2; Figure 3A) which were analyzed by PHASTER.^14^ The partial sequences of phage were inserted between the genes *yicC* and *dinD* (Part1) and genes *yfgI* and *guaA* (Part2; Figure 3B). The inserted partial sequences mainly contained some genes encoding hypothetical proteins (*hp*), domains of unknown function (DUFs), as well as genes encoding integrase and two new attachment sites (*attL* and *attR*) that integrate the phage genome into bacterial chromosomes. Besides, there were three main differences (Part 1, Part 2, and Part 3) between J-8 and the GZEC065 chromosome (Figure 4A). Three genes encoding hypothetical proteins were added downstream of the type II toxin-antitoxin system (*hipA-hipB*) in J-8 chromosome (Part 1), and the *yeeJ* gene in GZEC065 chromosome was truncated by genes encoding integrase, DNA-binding protein and hypothetical proteins (Part 2; Figure 4B). In addition, there were a large number of genes for *O*-antigen synthesis between *gndA* and *rfbC* genes, such as gene encoding glucosyltransferase, *wzx* and *wzy* (Part 3).

### Analysis of the genetic environment of *bla*_NDM-5_ at on IncX3-type plasmid

**Figure 5:**
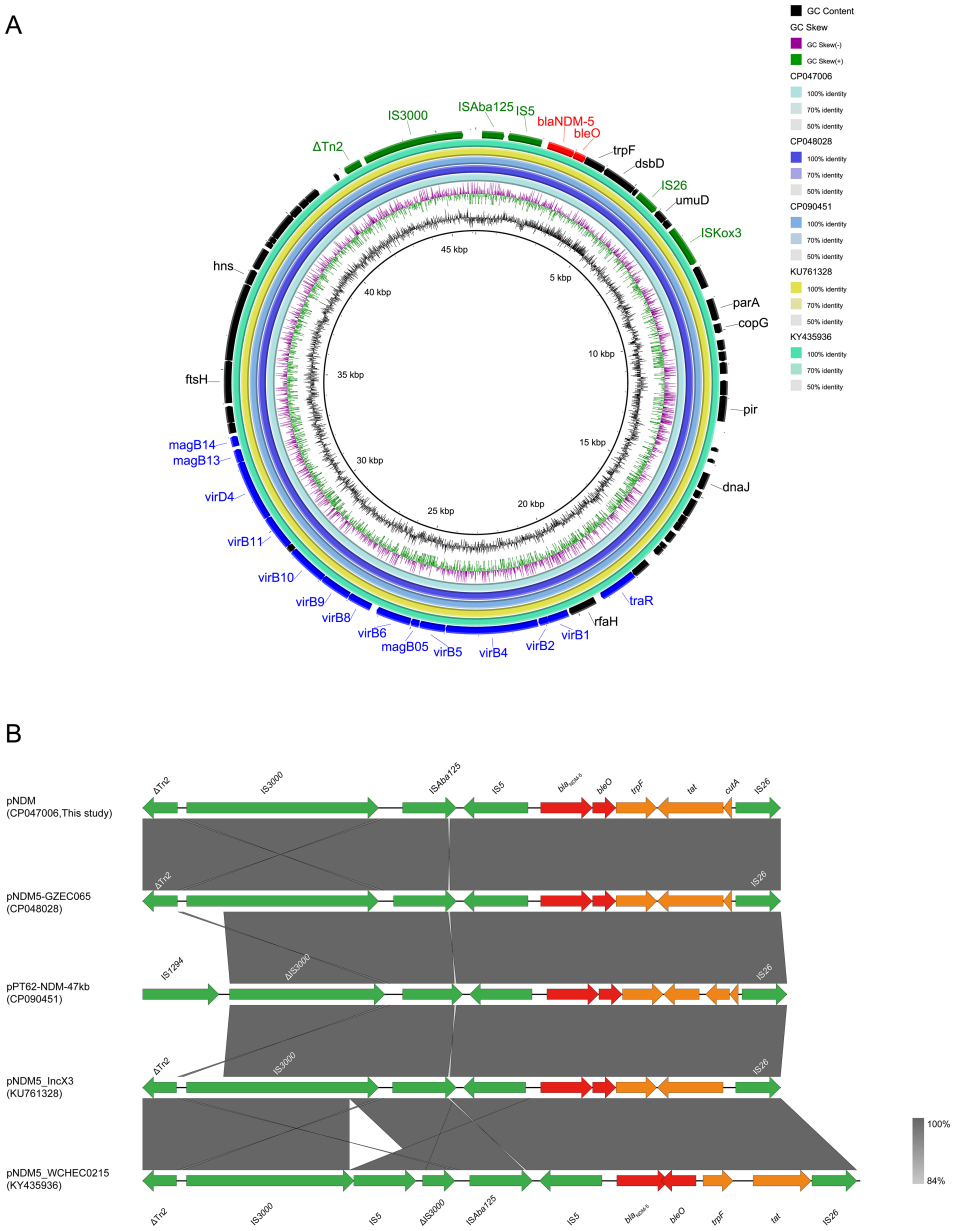
Comparative genomic analysis of *bla*_NDM-5_-carrying IncX3-type plasmid pNDM (CP047006, this study) with pNDM5-GZEC065 (CP048028), pPT62-NDM-47 kb (CP090451), pNDM5_IncX3 (KU761328) and pNDM5_WCHEC0215 (KY435936) **(A)**. The plasmid pNDM was used as a reference sequence, and dark colors represent other genes; blue colors represent relaxase, type IV secretion system (T4SS) and type IV coupling protein (T4CP); green colors represent insertion sequences (IS); red colors represent antimicrobial resistance (AMR) genes. Genetic environment of *bla*_NDM-5_ located on plasmid pNDM compared with pNDM5-GZEC065, pPT62-NDM-47 kb, pNDM5_IncX3 and pNDM5_WCHEC0215 **(B)**. Dark grey shading denotes regions of homology (>99% identity nucleotide identity). Orange colors represent other genes; green colors represent insertion sequence (IS) and transposon (Tn); red colors represent antimicrobial resistance (AMR) genes.

The *bla*_NDM-5_-carrying plasmid pNDM (CP047006) belonged to the IncX3 incompatibility group (Table 3). The backbone of this plasmid was highly similar to that of pNDM5-GZEC065 (CP048028; nucleotide coverage 100%; identity 99.99%), pPT62-NDM-47 kb (CP090451; nucleotide coverage 100%; identity 99.98%), pNDM5_IncX3 (KU761328; nucleotide coverage 100%; identity 99.99%) and pNDM5_WCHEC0215 (KY435936; nucleotide coverage 100%; identity 99.93%) as indicated by BLASTn analysis (Figure 5A). No other antimicrobial resistance genes were identified on the plasmid except for the genes *bla*_NDM-5_ and *bleO*. The genetic structure of *bla*_NDM-5_ in pNDM was IS*Aba125*-IS*5*-*bla*_NDM-5_-*bleO*-*trpF*-*tat*-*cutA*-IS26, and it was highly conserved in IncX3-type plasmids, with the *bla*_NDM-5_ gene in pNDM5-GZEC065, pPT62-NDM-47 kb and pNDM5_IncX3 (Figure 5B) being also similar. However, the transcription direction of *bleO* and *tat* genes, downstream of the *bla*_NDM-5_ gene in pNDM5_WCHEC0215, was opposite to those of other plasmids. The common mobile elements, IS*3000* and Tn*2* belonging to Tn*3* family, were also found on IncX3-type plasmid.

## Discussion

*Escherichia coli* containing both *mcr-1* and *bla*_NDM-5_ have been isolated from different sources around the worldwide, but those reported tend to be mainly located on plasmids. Subsequently, a few strains with *mcr-1* gene on the chromosome were reported, while those with coexisting chromosomal *mcr-1* gene and plasmid-bearing *bla*_NDM-5_ gene were rare. The sources were also mainly from human blood, ascites, stool and animal feces (Yu et al., 2016; Ho et al., 2018; Shen et al., 2018; Lu et al., 2022). Two MNPEC strains were isolated in this study, one from the liver of chickens with colibacillosis and the other from uterine lavage fluid of dairy cows with endometritis. Moreover, both of the strains showed XDR based on the definition provided by Magiorakos et al. (2012). Chromosomal *mcr-1* gene is conducive to its stable inheritance among populations and results in a dominant clone group with a new sequence type. In addition, the horizontal transfer of other AMR genes, such as *bla*_NDM-5_, *tet(X4)*, may further confer dire drug-resistant phenotypes to *mcr-1*-positive *E. coli*, thereby greatly promoting the emergence of XDR and even PDR-resistant bacteria.

MLST analysis of 56 MNPECs found that the dominant clones were CC156 (ST156), CC10 (ST10, ST167, ST48, ST744, and ST1602) and CC165 (ST189, ST1178, and ST165; Figure 1, data shown in Supplementary Table S1). Furthermore, MNPECs were widely distributed in China, especially in pig fecal samples, human stool and urine samples as well as the intestinal content of chicken. These results indicated that coexisting *mcr-1* and *bla*_NDM-5_ genes in *E. coli* were still quite common in animals and humans in China. The emergence of *mcr-1* gene in *E. coli* is linked to the use of polymyxin. In 2017, colistin, as a feed additive in animals, had been banned in China, and the subsequent implementation of colistin withdrawal policy reduced colistin resistance in both animals and humans (Wang et al., 2020). However, the data collected in this study were all from published papers and may not reflect the real epidemic situation of MNPEC in China. Nevertheless, the surveillance of drug resistance and the standardized use of antimicrobials remain still effective means to curb the spread of bacterial drug resistance.

*mcr-1*-positive *E. coli* ST156 was previously isolated only from the patients’ blood culture and ducks’ rectal swab (Yang et al., 2016; Rossi et al., 2017; Lin et al., 2020), which exhibited MDR or XDR including meropenem and colistin. In this study, MNPECs were mainly distributed in *E. coli* ST156 (Figure 1A) and two MNPECs isolated from diseased chicken and dairy cow, also belonged to ST156 and exhibited XDR, including resistance to carbapenems and polymyxins. The coexistence of multiple AMR genes, including *mcr-1*, *bla_CTX-M-55_* and *bla*_NDM-5_ in J-8 and N-14 conferred their multi-resistance phenotype. At the same time, most of the reports regarding the coexisting *bla*_NDM-5_ and *mcr-1* genes within *E. coli* ST156 were mainly located on plasmids (Sun et al., 2016; Yang et al., 2016; Kong et al., 2017). Indeed, while the coexistence of chromosomal *mcr-1* and plasmid-bearing *mcr-1 gene* within *E. coli* ST156 was rare, the strains originated mainly from human (Yu et al., 2016; Lin et al., 2020). *Escherichia coli* ST156 has been shown to be associated with different ESBL genes and humans (Pan et al., 2013; Cortes-Cortes et al., 2016; Wang et al., 2020), in this study, with previous studies finding that ST156 also contained the *bla*_CTX-55_ gene and largely isolated from animal source (Liu et al., 2021b). Overall, the findings indicated the fact that ST156 had spread among humans and animals in China. In addition, the coexistence of *mcr-1*, *bla*_NDM-5_ and ESBLs genes in *E. coli* ST156 implied that ST156 may become a new reservoir of AMR genes and the most common transmission was a clone group as ST10.

The *mcr-1* gene is present in various plasmid types, of which IncI2-, IncX4- and IncHI2-type ones are the most common carriers (Matamoros et al., 2017). This gene was embedded in a ~ 2.6-kb *mcr-1*-*pap2* cassette, with the insertion sequence IS*Apl1* flanking the gene being present in various forms, such as IS*Apl1*-*mcr-1*-*pap2*-IS*Apl1*(Tn*6330*), IS*Apl1*-*mcr-1*-*pap2*, *mcr-1*-*pap2* (Li et al., 2016; Yang et al., 2016; Li et al., 2017). Moreover, the *mcr-1* gene with one IS*Apl1* copy or without IS*Apl1* copies were formed by the deletion of IS*Apl1* from the ancestral Tn*6330*, with its transposition mechanism being the formation of a circular intermediate (3,679 bp, IS*Apl1*-*mcr-1*-*pap2*; Li et al., 2017; Snesrud et al., 2018). However, the *mcr-1* gene located on the chromosome was mainly embedded in an intact composite transposon Tn*6330* or flanked by two copies of IS*Apl1* (Yu et al., 2016; Sun et al., 2018; Lin et al., 2020; Shen et al., 2020; Yamaguchi et al., 2020). The results also found that the *mcr-1* gene on the chromosome was located as an intact Tn*6330*, which was consistent with previous reports. Besides, since the analysis of chromosomes carrying *mcr-1* gene has not been reported previously, it was found that the differences between chromosomes were the partial sequence of phage integrated in host genome as well as the genes associated with *O*-antigen synthesis through comparative genomics analysis.

IncX3-type plasmids have a narrow-host-range, especially for *Enterobacteriaceae*, which has strong transferability, stability and low fitness cost, amongst others (Wu et al., 2019; Guo et al., 2022). The *bla*_NDM-5_ gene was mainly located on a 46-kb self-transmissible IncX3-type plasmid, which was the most common vector of *bla*_NDM-5_ (Wu et al., 2019). In this study, the *bla*_NDM-5_ was located on IncX3-type plasmid pNDM and could be transferred to the recipient strain along with the plasmid (Table 2). Meanwhile, the backbone structure of *bla*_NDM-5_-carrying the IncX3-type plasmid was highly conserved, including the IS*Ab125*-IS*5*-*bla*_NDM-5_-*bleO*-*trpF*-*tat*-*cutA*-IS*26* gene cassette, the T4SS systems, the plasmid replication/maintenance genes and mobile elements IS*3000*, Tn*2* (Figure 5). Moreover, *bla*_NDM-5_ gene was mostly located on the Incx3-type plasmid in MNPECs, regardless of the location of the *mcr-1* gene on the chromosome or plasmid (Yu et al., 2016; Kong et al., 2017; Ho et al., 2018; Kuang et al., 2022; Wang et al., 2022). This indicated that IncX3-type plasmid was the dominant vector for carrying *bla*_NDM-5_ and disseminate the carbapenem resistance in MNPECs.

## Conclusion

Nine MNPECs displayed XDR by being resistant to antimicrobials that are commonly used against *Enterobacteriaceae*, with CC156 (ST156) being the new dominant clones in MNPECs. *bla*_NDM-5_ located on the plasmid was successfully transferred to C600 which then showed extensive resistance to β-lactam antibiotics. In addition, the conjugative transfer of the *mcr-1* gene, located on the chromosome, failed. J-8 and N-14 strains contained point mutations associated with quinolone resistance as well as more than three types of AMR genes, especially the chromosomal *mcr-1* gene and the *bla*_NDM-5_ gene located on the IncX3-type plasmid within the same strain. This characteristic conferred extensive drug resistance to the strains. The genetic structure of *mcr-1*, located on the chromosome, was an intact Tn*6330* and was highly conserved in MNPECs. Moreover, the differences between chromosomes were the additional partial sequence of phage integrated in host genome and the different genes associated with *O*-antigen synthesis. IncX3-type plasmid was the dominant vector for carrying *bla*_NDM-5_ and disseminate the carbapenem resistance in MNPECs.

## Data availability statement

The datasets presented in this study can be found in online repositories. The names of the repository/repositories and accession number(s) can be found in the article/Supplementary material.

## Author contributions

PC and ZL: conceptualization. ZL: methodology, software, formal analysis, data curation, and visualization. PC, YX, GY, and YS: validation. LT, PC, YL, XG, and LM: investigation. JG: resources. ZL and PC: writing—original draft preparation. PC and YL: writing—review and editing. PC: supervision, project administration, and funding acquisition. All authors contributed to the article and approved the submitted version.

## Funding

This research was supported by National Key Research and Development Program Project in China (grant number 2017YFD0501400); 2021 Joint project of Medical Science and Technology Public Relations Plan of Henan Province (Grant number LHGJ20210373).

## Conflict of interest

PC was employed by Shandong Xindehui Biotechnology Company Ltd.

The remaining authors declare that the research was conducted in the absence of any commercial or financial relationships that could be construed as a potential conflict of interest.

## Publisher’s note

All claims expressed in this article are solely those of the authors and do not necessarily represent those of their affiliated organizations, or those of the publisher, the editors and the reviewers. Any product that may be evaluated in this article, or claim that may be made by its manufacturer, is not guaranteed or endorsed by the publisher.

## Footnotes

^1^ https://www.ncbi.nlm.nih.gov/pathogens/refgene/#NDM

^2^http://enterobase.warwick.ac.uk/species/ecoli/ [Accessed October 19, 2022].

^3^https://rast.nmpdr.org/rast.cgi [Accessed October 19, 2022].

^4^https://cge.cbs.dtu.dk/services/PlasmidFinder/ [Accessed October 19, 2022].

^5^https://cge.cbs.dtu.dk//services/ResFinder/ [Accessed October 19, 2022].

^6^https://card.mcmaster.ca/analyze/rgi [Accessed October 19, 2022].

^7^https://cge.cbs.dtu.dk/services/VirulenceFinder/ [Accessed October 19, 2022].

^8^https://cge.cbs.dtu.dk/services/FimTyper/ [Accessed October 19, 2022].

^9^https://tool-mml.sjtu.edu.cn/oriTfinder/oriTfinder.html [Accessed October 19, 2022].

^10^https://www-is.biotoul.fr/blast.php [Accessed October 19, 2022].

^11^http://integrall.bio.ua.pt/?search [Accessed October 19, 2022].

^12^https://cge.cbs.dtu.dk/services/MobileElementFinder/ [Accessed October 19, 2022].

^13^http://clermontyping.iame-research.center/ [Accessed October 19, 2022].

^14^http://phaster.ca/ [Accessed October 19, 2022].
